# The First Whole Genome Sequencing of *Sanghuangporus sanghuang* Provides Insights into Its Medicinal Application and Evolution

**DOI:** 10.3390/jof7100787

**Published:** 2021-09-22

**Authors:** Ji-Hang Jiang, Sheng-Hua Wu, Li-Wei Zhou

**Affiliations:** 1 State Key Laboratory of Mycology, Institute of Microbiology, Chinese Academy of Sciences, Beijing 100101, China; jiangjh@im.ac.cn; 2 Department of Biology, National Museum of Natural Science, Taichung 404, China; shwu@mail.nmns.edu.tw

**Keywords:** *Basidiomycota*, mating system, secondary metabolites, species diversification, wood-inhabiting macrofungi

## Abstract

*Sanghuangporus* is a medicinal macrofungal genus typified by *S. sanghuang*, the very species utilized in traditional Chinese medicines by Chinese ancient people. To facilitate the medicinal application of *S. sanghuang*, we, for the first time, perform its genome sequencing and analyses from a monokaryon strain. A 33.34 Mb genome sequence was assembled to 26 contigs, which lead to the prediction of 8278 protein-coding genes. From these genes, the potential biosynthesis pathway of sesquiterpenoids was, for the first time, identified from *Sanghuangporus*, besides that of triterpenoids. While polysaccharides are the main medicinal metabolites in *S. sanghuang*, flavonoids are especially abundant medicinal metabolites comparing with other medicinal macrofungal groups. From the genomic perspective, *S. sanghuang* has a tetrapolar heterothallic mating system, and has its special nutritional strategy and advantageous medicinal properties compared with *S. baumii* and *S. vaninii*. A phylogenomics analysis indicates that *Sanghuangporus* emerged 15.39 million years ago and *S. sanghuang* has a closer phylogenetic relationship with *S. baumii* than *S. vaninii*. However, *S. sanghuang* shares a higher region of synteny and more orthologous genes, including carbohydrate-active enzymes with *S. vaninii* than *S. baumii*. A comparative genomics analysis with *S. baumii* and *S. vaninii* indicates that species diversification within *Sanghuangporus* may be driven by the translocation and translocation plus inversion of genome sequences, while the expansion and contraction of gene families may contribute to the host specificity of *Sanghuangporus* species. In general, the genome sequence of *S. sanghuang* provides insights into its medicinal application and evolution.

## 1. Introduction

As a group of medicines in traditional Chinese medicines, macrofungi have been utilized by Chinese ancient people for 6800 years [1] and were recorded in *Shen Nong Material Medica* 2000 years ago [2]. Of the myco-medicinal resources, certain fungal species, such as “Donchong Xiacao” (*Ophiocordyceps sinensis*), “Lingzhi” (*Ganoderma lingzhi*) and “Fuling” (*Wolfiporia cocos*) are, nowadays, well-known and commercially sold around the world, especially in China. In addition to these fungi, “Sanghuang” (*Sanghuangporus* spp.) has attracted more and more attentions from folklore, scientific and industrial fields [3].

“Sanghuang” is represented by a wood-inhabiting macrofungal genus *Sanghuangporus* belonging to *Hymenochaetaceae*, *Basidiomycota* [4]. Its main medicinal functions are composed of antitumor, antioxidant, anti-inflammation and immunomodulation, for which polysaccharides, polyphenols, pyrones and terpenes are responsible [3,5]. Fifteen species are accommodated in *Sanghuangporus* [6,7], and three species, viz. *S. baumii*, *S. sanghuang* and *S. vaninii*, exclusively inhabiting *Syringa*, *Morus* and *Populus*, respectively, are commonly involved in medicinal application [3]. Of them, *S. sanghuang* is considered to be the very species recorded in ancient books of traditional Chinese medicines and utilized by Chinese ancient people [4,8].

With the advance of DNA sequencing technologies, medicinal studies on wood-inhabiting macrofungi have gradually relied on genome sequences besides those continuously on the metabolites themselves. *Ganoderma lingzhi* [9,10], *Taiwanofungus camphoratus* [11,12], *Hericium erinaceus* [13,14], *Auricularia heimuer* [15] and *Russula griseocarnosa* [16] have been used for whole genome sequencing. These genomic analyses help to understand the medicinal properties, biosynthetic pathways, mating types and trophic modes of these wood-inhabiting macrofungi, which further facilitate their medicinal utilization and industrial development.

Regarding species in the genus *Sanghuangporus*, three whole genome sequences are available: the first one represents *S. baumii* and was directly uploaded to the National Center for Biotechnology Information (NCBI; https://www.ncbi.nlm.nih.gov/bioproject/PRJNA304358/) without a detailed analysis; the second one was well analyzed [17], but it was sequenced from *S. vaninii* instead of the wrongly stated *S. sanghuang* [7,18]; the third one, also, with detailed analyses was wrongly identified to be sequenced from *Phellinus* *givus* [19], but is actually from *S. vaninii* [7]. Two of three available whole genomes of *Sanghuangporus* are attributed to inaccurate species due to misidentifications following a bad taxonomic practice [7]. This phenomenon could block the utilization of unique medicinal properties of each *Sanghuangporus* species and, thus, delay a further industrial development when applying for permissions of commercial products [20].

In addition to *S. baumii* and *S. vaninii*, another commonly involved species of *Sanghuangporus* in medicinal application, viz. *S. sanghuang*, has never been genome sequenced, even though this species is more important in the cultural history of traditional Chinese medicines [8]. Previously, *S. sanghuang* has been evidenced to possess better medicinal properties of antitumor, antioxidant and anti-inflammation than *S. baumii* and *S. vaninii* [21]. However, the large-scale cultivation of *S. sanghuang* is not successful when comparing it with that of *S. vaninii*.

To facilitate the medicinal utilization and industrial development of *S. sanghuang* from the genomic perspective, we generated the first genome sequence from a monokaryotic strain of this fungal species following an exact species identification. Besides information for medicinal application, the genomic analyses, especially via a comparison with genomes of other related fungal species, also revealed evolutionary and speciated dynamics of *Sanghuangporus*.

## 2. Materials and Methods

### 2.1. Strain Culture and DNA Isolation

The monokaryotic strain MS2 of *Sanghuangporus sanghuang* was isolated from wild fruiting bodies collected from Zhejiang Province, China, in May 2018. The mycelia of MS2 were harvested after growing on the sterile cellophane covering potato dextrose agar (PDA) plate culture medium with the addition of 25% sawdust filtrate at 28 °C for 5–7 days. Genomic DNA was extracted from the harvested mycelia with the SDS method [22]. The enriched genomic DNA was detected by the agarose gel electrophoresis and quantified by Qubit^®^ 2.0 Fluorometer (Thermo Scientific, Waltham, MA, USA).

### 2.2. Species Identification

The ITS region of MS2 was amplified from the genomic DNA using the primers ITS5 and ITS4 [23]. The PCR procedure was as follows: initial denaturation at 95 °C for 3 min, followed by 34 cycles at 94 °C for 40 s, 57 °C for 45 s and 72 °C for 1 min, and a final extension at 72 °C for 10 min. The PCR product was sequenced with the primer ITS5 at the Beijing Genomics Institute, Beijing, China. The generated ITS sequence was deposited in GenBank (https://www.ncbi.nlm.nih.gov/genbank/; MZ255059) and was used for species identification via BLAST search according to the latest species delimitation in Shen et al. [9].

### 2.3. Genome Sequencing and Assembly

PacBio Sequel and Illumina NovaSeq PE150 platforms were used for whole genome sequencing at the Beijing Novogene Bioinformatics Technology Co., Ltd. (Beijing, China) Library for single-molecule real-time (SMRT) sequencing in the PacBio Sequel platform was constructed with an insert size of 20 kb using the SMRTbell™ Template kit version 1.0. The library quality was assessed by Qubit^®^ 2.0 Fluorometer (Thermo Scientific) and the insert fragment size was detected by Agilent 2100 Bioanalyzer (Agilent Technologies, Santa Clara, CA, USA).

Regarding the Illumina NovaSeq PE150 platform, sequencing library of 350 bp was generated from 1 μg DNA using NEBNext^®^ Ultra™ DNA Library Prep Kit for Illumina (NEB, Ipswich, MA, USA) following manufacturer’s recommendation. The library was analyzed for size distribution by Agilent 2100 Bioanalyzer (Agilent Technologies).

After filtering low-quality reads (less than 500 bp), the clean data from the PacBio Sequel platform were preliminary assembled using SMRT Link version 5.0.1. The long reads were selected (more than 6 kb) as the seed sequence, and the other shorter reads were aligned to the seed sequence using BLASR version 5.3.5 [24]. Finally, the arrow algorithm was used to correct and count the variant sites in the initial genome sequence using the variant Caller module of the SMRT Link.

### 2.4. Gene Prediction and Annotation

Protein-coding gene models were de novo predicted using Augustus version 3.3.3 [25] due to lack of reference genome. The interspersed repetitive sequences were predicted using RepeatMasker (http://www.repeatmasker.org/, accessed on 22 April 2020). The tandem repeats were analyzed using Tandem Repeats Finder version 4.0 [26]. tRNA genes were predicted using tRNAscan-SE version 3.0 [27].

Gene functions were predicted with the references of nine databases, viz. Gene Ontology (http://geneontology.org/, accessed on 27 February 2020), Kyoto Encyclopedia of Genes and Genomes (KEGG) (https://www.kegg.jp/, accessed on 27 February 2020), Eukaryotic Orthologous Groups (https://www.creative-proteomics.com/services/kog-annotation-analysis-service.htm, accessed on 27 February 2020), Non-Redundant Protein Database (NR) (https://www.ncbi.nlm.nih.gov/protein/, accessed on 27 February 2020), Transporter Classification Database (http://www.tcdb.org, accessed on 27 February 2020), Fungal Cytochrome P450 Database (http://p450.riceblast.snu.ac.kr/cyp.php, accessed on 27 February 2020), Swiss-Prot (https://www.uniprot.org/, accessed on 27 February 2020), Pfam (http://pfam.xfam.org/, accessed on 27 February 2020) and Carbohydrate-Active enZYmes (CAZymes) Database (http://www.cazy.org/, accessed on 27 February 2020). All predicted coding genes were aligned with these nine databases using DIAMOND version 2.0.2 [28] with the cut-off values of E-value no more than 1 × 10^−5^, identity not less than 40% and coverage not less than 40%. When a single gene retrieved more than one result meeting the cut-off values from databases, this gene was annotated following the result with the best score.

antiSMASH program version 2.0.2 was employed to predict the gene clusters of secondary metabolites with default parameters [29].

### 2.5. Genomic Structure of the Mating-Type (MAT) Locus

In addition to homeodomain transcription factor genes (*HD*) and pheromone/receptor genes identified based on the databases of NR, Swiss-Prot and Pfam, the genes that flank the *MAT* locus were further identified via BLAST search. The following sequences were used as queries: XP_007265184.1 from *Fomitiporia mediterranea*, XP_003038723.1 from *Schizophyllum commune*, XP_036634433.1 from *Pleurotus ostreatus*, XP_007325204.1 from *Agaricus bisporus* and XP_008032819.1 from *Trametes versicolor* for identifying mitochondrial intermediate peptidase gene (*mip*), while XP_009540982.1 from *Heterobasidion irregular*, XP_006454075.1 from *Agaricus bisporus* and XP_001829147.2 from *Coprinopsis cinerea* for β-flanking gene (*β-fg*).

### 2.6. Phylogenomics Analysis

To explore the evolutionary dynamics of *S. sanghuang*, the genome sequences of additional 22 fungal species were downloaded from NCBI for phylogenomics analysis (Table 1).

Single-copy orthologous genes from the 23 fungal species were inferred using OrthoFinder version 2.3.12 [30] with mafft option for subsequent multiple sequence alignment. On the basis of the resulting alignment, maximum likelihood tree was constructed using RAxML version 8.2.12 [31] with GTR + I + G model. Two ascomycetous species, viz. *Neurospora crassa* and *Tuber melanosporum*, were selected as the outgroup taxa of another 21 basidiomycetous species (Table 1). Statistical support values were obtained using nonparametric bootstrap with 1000 replicates. Divergence times of the 23 fungal species were estimated using the mcmctree program of PAML version 4.4 [32] with the following parameters: ndata = 1; seqtype = 2; burnin = 1,000,000; sampfreq = 10; nsample = 500,000. Four calibration points were selected as normal priors to restrain the age of the nodes, including the divergence time of 227–277 million years ago (Mya) between *Rickenella* and *Sanghuangporus*, 187–236 Mya between *Trametes* and *Heterobasidion*, 110–201 Mya between *Pleurotus* and *Schizophyllum*, and 626–806 Mya between *Agaricus* and *Neurospora* [33].

Expansion and contraction of gene families were determined using CAFE version 4.2.1 [34] with the following parameters: a cut-off *p*-value of 0.05; number of random samples = 1000; the lambda value to calculate birth and death rates.

### 2.7. Comparative Genomics Analysis

To explore the dynamics of speciation in *Sanghuangporus*, the genome sequences of *S. baumii*, *S. sanghuang* and *S. vaninii* were aligned in pairs (Table 1) using MCScan (Python version) [35]. Based on the resulting blocks, genomic synteny map among the three species was drawn using jcvi package in Python 3 [35], and genomic blocks of collinearity, translocation, inversion and translocation plus inversion between *S. sanghuang* and *S. baumii* or *S. vaninii* were analyzed using MUMmer 4 (https://sourceforge.net/projects/mummer/, accessed on 14 December 2020).

To identify the differences and similarities of medicinal application in *Sanghuangporus* with a uniform standard, the genome sequences of *S. baumii* and *S. vaninii* were reannotated using the same pipeline as that of *S. sanghuang*. Then, genome structure and protein-coding genes related to medicinal utilization were compared among these three species. In addition, the numbers of genes encoding various families of CAZymes in these three species were clustered in heatmap using TBtools version 1.9857 [36] with option of log scale.

## 3. Results

### 3.1. Species Identity

Following the latest taxonomic standard of *Sanghuangporus* [7], the strain MS2 was identified to *S. sanghuang* using an ITS barcoding sequence (congruent with JN794061 from the holotype of *S. sanghuang*).

### 3.2. Genome Sequence Assembly and Annotation

**Figure 1:**
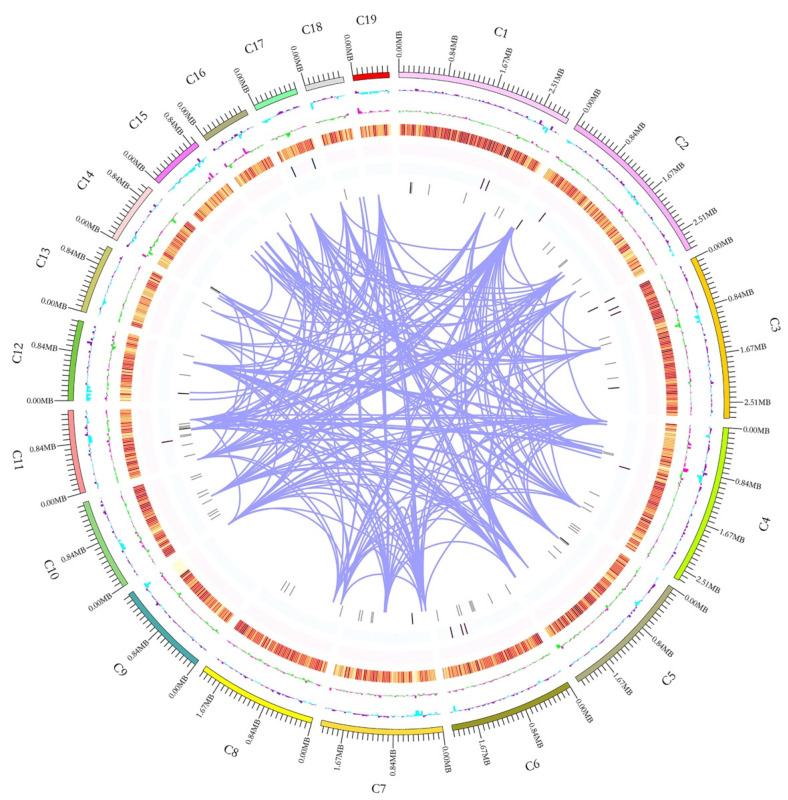
Characteristics of the de novo assembly genomic features of *Sanghuangporus sanghuang*. From the outside to the inside are: 1. Contigs (>1 Mb in length); 2. GC content: calculated as the percentage of G + C in 1 kb non-overlapping windows. The inward blue part represents the GC content in the region lower than the average genome GC content, while the outward purple part represents the opposite; 3. GC skew: calculated as the percentage of (G − C)/(G + C) in 1 kb non-overlapping windows. The inward green part represents G/C < 1, while the outward pink part represents G/C > 1; 4. Gene density: four circles starting from the orange color to inside, respectively, represent the numbers of coding genes, rRNA, snRNA and tRNA in 1 kb non-overlapping windows. The intensity of the color positively correlates with gene density; 5. Genome duplication: regions sharing more than 90% sequence similarity over 8 kb are connected by purple lines.

The first whole genome sequence (GenBank accession number: JAHSQH000000000) of *S. sanghuang* was generated from the monokaryotic strain MS2 using PacBio Sequel and Illumina NovaSeq PE150 platforms. The 33.34 Mb genome sequence was assembled from 5589 Mb raw data (168× genome coverage) and to 26 contigs with a GC content of 48.04% (Table 2). Of the 26 contigs, the longest one was 2.98 Mb in length, while the N50 length was 2.06 Mb (Table 2). The GC skew did not present an obvious distribution pattern in the whole genome (Figure 1). A K-mer analysis with a depth of 23.24 showed a 0.03% heterozygous rate of the genome, which further confirmed that MS2 is a monokaryon strain. All the above information indicates the high quality of the genome sequence assembly of *S. sanghuang*.

A total of 8278 protein-coding genes were predicted with an average gene length of 1597 bp (Table 2, File S1), while 104 non-coding RNAs (ncRNAs) were predicted, accounting for 2.91% of the whole genome sequence. The total length of repetitive sequences was 1.25 Mb (Table 2) with the tandem repeat sequence and the interspersed repeated sequence, respectively, accounting for 0.85% and 2.90% of the whole genome sequence. The length of the transposable elements was 0.96 Mb with long terminal repeats (LTR) and non-LTR, respectively, accounting for 2.72% and 0.17% of the whole genome sequence.

### 3.3. Terpenoid Biosynthesis

Fourteen key enzymes involved in terpenoid backbone biosynthesis via the mevalonate (MVA) pathway were identified from *S. sanghuang* (Figure S1). Of the 14 key enzymes, each of farnesyl diphosphate synthase and protein farnesyltransferase subunit beta was encoded by a double-copy gene, while the other 12 enzymes were encoded by single-copy genes (Table S1). In addition to the 14 key enzymes in the MVA pathway, genes indirectly related to terpenoid biosynthesis were also identified: one gene (ID: A8109) encoding lanosterol synthase, three genes (IDs: A4471, A1626 and A2280) involved in the biosynthesis of sesquiterpenoids and triterpenoids (Figure S2), and six genes (IDs: A6069, A6073, A6078, A6085, A7294 and A7998) involved in the biosynthesis of trichodiene synthases.

### 3.4. Polysaccharide Biosynthesis

A total of 22 enzymes encoded by 41 genes and involved in the biosynthesis of polysaccharides (starch and sucrose metabolism) were identified from *S. sanghuang* (Figure S3, Table S2). Most of these enzymes are encoded by single- or double-copy genes, while the beta-glucosidase and the cellulose 1,4-beta-cellobiosidase are encoded by five- and seven-copy genes, respectively. In addition, 12 enzymes encoded by 34 genes were identified to participate the biosynthesis of uridine diphosphate glucoses, the precursor of glucans (Table S3).

### 3.5. Flavonoid Biosynthesis

No key enzyme directly related to the pathway of flavone and flavonol biosynthesis was identified from *S. sanghuang.* However, eight putative genes involved in the biosynthesis of flavonoid compounds were annotated, including isoflavonoid 7-O-beta-apiosyl-beta-glucosidase, chalcone 4′-O-beta-glucosyltransferase, chalcone-flavanone isomerase and flavonol L-rhamnosyltransferase (Table S4).

### 3.6. CAZyme

From *S. sanghuang*, a total of 346 genes were assigned to encode the six classes of CAZymes, including 38 genes encoding carbohydrate-binding modules (CBMs), 23 carbohydrate esterases (CEs), 162 glycoside hydrolases (GHs), 62 glycosyltransferases (GTs), 9 polysaccharide lyases (PLs) and 52 auxiliary activities (AAs) (Table 3). Among the six classes, GHs encoded by most genes were mainly involved in the degradation of celluloses (GH5, GH6, GH7 and GH12), hemicelluloses (GH10 and GH43), pectins (GH28 and G105), chitins (GH18) and starches (GH31 and GH133). The families each encoded by ten or more genes included GH16 (19), GH5 (18), AA2 (12), GH18 (11), GT2 (11) and AA1 (10).

### 3.7. Cytochromes P450 (CYPs)

A total of 121 genes were annotated to encode CYPs in *S. sanghuang* (Table 3). Of these genes, 12 were classified to encode CYPs belonging to the Cytochrome P450 class, 1 to the E-class P450, CYP2D class, 66 to the E-class P450, group I class, 8 to the E-class P450, group IV class, 3 to P450, the CYP52 class and 6 to the pisatin demethylase-like class, while 25 genes were determined to encode CYPs not belonging to any known class. The E-class P450, group I class encoded by the most genes is involved in the oxidation-reduction reactions, while the Cytochrome P450 class encoded by the second most genes is usually responsible for the signal transduction of metabolic process. The CYPs encoded by the 25 genes and not belonging to any known class were predicted to participate in several kinds of secondary metabolic processes, such as the biodegradation of xenobiotics, carbohydrate metabolism and biosynthesis of antibiotics.

### 3.8. Gene Cluster

Sixteen gene clusters were predicted from *S. sanghuang*. Of them, ten were identified to encode terpene synthases (TS), four to iterative type I polyketide synthases (T1PKS), one to nonribosomal peptide synthetases (NRPS) and one to other molecules (Table 3).

### 3.9. MAT Locus

Three genes encoding HD were identified as the *MAT-A* locus via genome annotation on the basis of the NR (IDs: A5973 and A7529) and Pfam (ID: A6629) databases. At the amino acid level, A5973 had the highest similarity of 52.17% to homeodomain protein in *Volvariella volvacea*, while A6629 and A7529 had the highest similarities of 91.77% and 99.72%, respectively, to the hypothetical protein A7U60_g4430 and the homeobox-domain-containing protein in *S. baumii*. Moreover, the flanking genes were identified only around A7529 on contig eight; the *β-fg* gene in the upstream of A7529 had a similarity of 51.43% to that in *A. bisporus*, while the *mip* gene in the downstream of A7529 had a similarity of 80.25% to that in *F. mediterranea*. This phenomenon indicated that A5973 and A6629 may not be involved in the mating system. Two adjacent pheromone receptor-coding genes (IDs: A3780 and A3781) were identified on contig two of the genome of *S. sanghuang*. These two genes had high similarities to the STE3 gene encoding α-factor pheromone receptor in *S. baumii* (98.38% and 98.76%, respectively) and *F. mediterranea* (73.02% and 55.91%, respectively). In addition, a kinase-coding gene CLA4 (ID: A3784) related to *MAT-B* locus was identified to be close to A3780 and A3781 at a distance of 6.97 kb on contig two. This further confirmed A3780 and A3781 as the *MAT-B* locus in *S. sanghuang*. No pheromone precursor-coding gene was identified from the genome of the monokaryon strain MS2.

### 3.10. Phylogenomics

**Figure 2:**
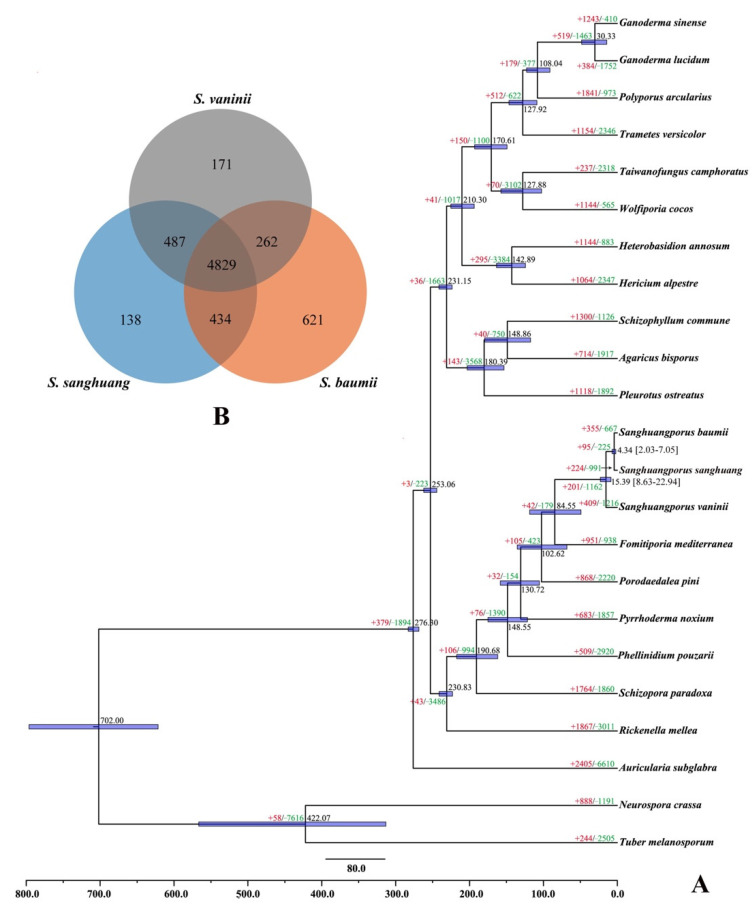
(**A**). Maximum clade credibility tree inferred from 429 single-copy orthologous genes. All nodes received full bootstrap support. The divergence time is labeled as the mean crown age for each node, while the 95% highest posterior density is also given within the *Sanghuangporus* clade. The numbers of gene family expansion and contraction in each species are labeled after plus (in red color) and minus (in green color) symbols, respectively. (**B**). Venn diagram of orthologous groups in *Sanghuangporus baumii*, *S. sanghuang* and *S. vaninii*.

A total of 1513 orthologous groups, including 429 single-copy genes, were identified from all 23 studied fungal species. The phylogenomics tree inferred from an alignment of the 429 single-copy orthologous genes with 673,947 characters of amino acid residues delimited phylogenetic relationships among the 23 species with full bootstrap support (Figure 2A). Besides three species in *Sanghuangporus*, other well-recognized medicinal fungal species, viz. *Ganoderma lucidum*, *G. sinense*, *S. commune*, *T. camphoratus* and *W. cocos*, were also phylogenetically separated. *Sanghuangporus* was estimated to emerge in a mean crown age of 15.39 Mya with a 95% highest posterior density (HPD) of 8.63–22.94 Mya. Of the species in *Sanghuangporus*, *S. sanghuang* and *S. baumii*, occurring in a mean crown age of 4.34 Mya with a 95% HPD of 2.03–7.05 Mya, had a closer phylogenetic relationship.

In the evolutionary process of the 23 sampled fungal species, the gene family contraction occurred more common than the gene family expansion (Figure 2A). Regarding *Sanghuangporus*, 355, 224 and 409 gene families had, respectively, expanded in *S. baumii*, *S. sanghuang* and *S. vaninii*, corresponding to 667, 991 and 1216 gene families being contracted. Of these gene families, 15 (8 expanded and 7 contracted), 13 (all expanded) and 11 (5 expanded and 6 contracted) in *S. baumii*, *S. sanghuang* and *S. vaninii*, respectively, experienced a rapid evolution.

### 3.11. Comparative Genomics

**Figure 3:**
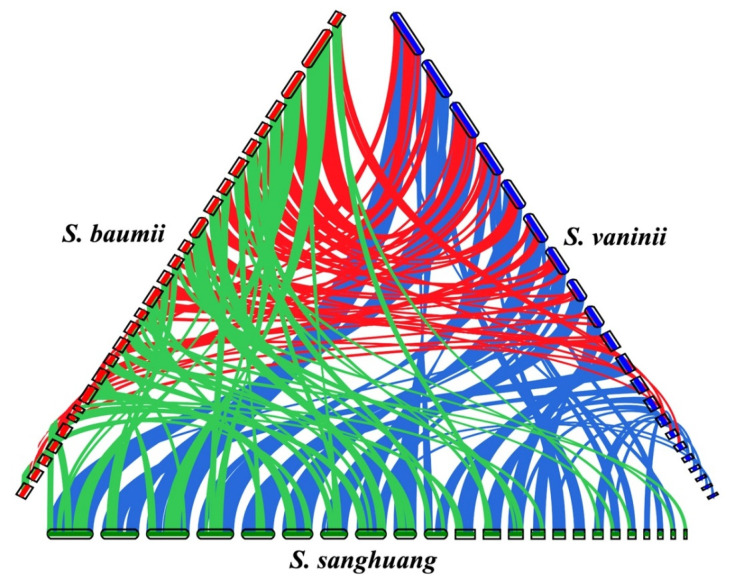
Genomic synteny of *Sanghuangporus baumii*, *S. sanghuang* and *S. vaninii*. Shared blocks of synteny are linked by red lines between *S. baumii* and *S. vaninii* (62.77% syntenic genes in 95 blocks), green lines between *S. baumii* and *S. sanghuang* (65.51% syntenic genes in 96 blocks), and blue lines between *S. sanghuang* and *S. vaninii* (85.04% syntenic genes in 57 blocks).

The genome sizes among the three species of *Sanghuangporus* were quite similar, but the number of predicted protein-coding genes was much higher in *S. vaninii* than in another two species (Table 3). The whole genome sequence of *S. sanghuang* had a higher similarity to that of *S. baumii* (93.26%) than to that of *S. vaninii* (80.96%). However, while 4829 orthologous groups were identified from the three species of *Sanghuangporus*, *S. sanghuang* shared more orthologous groups with *S. vaninii* (487) than *S. baumii* (434) and *S. baumii* and *S. vaninii* shared the least orthologous groups (262) (Figure 2B). In addition, *S. baumii* had the most unique orthologous groups (621) (Figure 2B). Similarly, the shared synteny between *S. sanghuang* and *S. vaninii* was the highest among the three species (Figure 3). Of the shared synteny between *S. sanghuang* and each of the other two species, the region of translocation and translocation plus inversion was much higher than that of the collinearity accounting only for a small proportion of the whole genome sequence (Figure S4).

Among the three species of *Sanghuangporus*, the number of gene clusters involved in the synthesis of TS was similar, while *S. sanghuang* had the highest and the lowest numbers of gene clusters involved in the syntheses of T1PKS and NRPS, respectively (Table 3). From the perspective of biosynthetic pathways, the three species of *Sanghuangporus* had a comparable number of genes involved in the pathways of terpenoid backbones, polysaccharides and flavonoids (Table 3).

**Figure 4:**
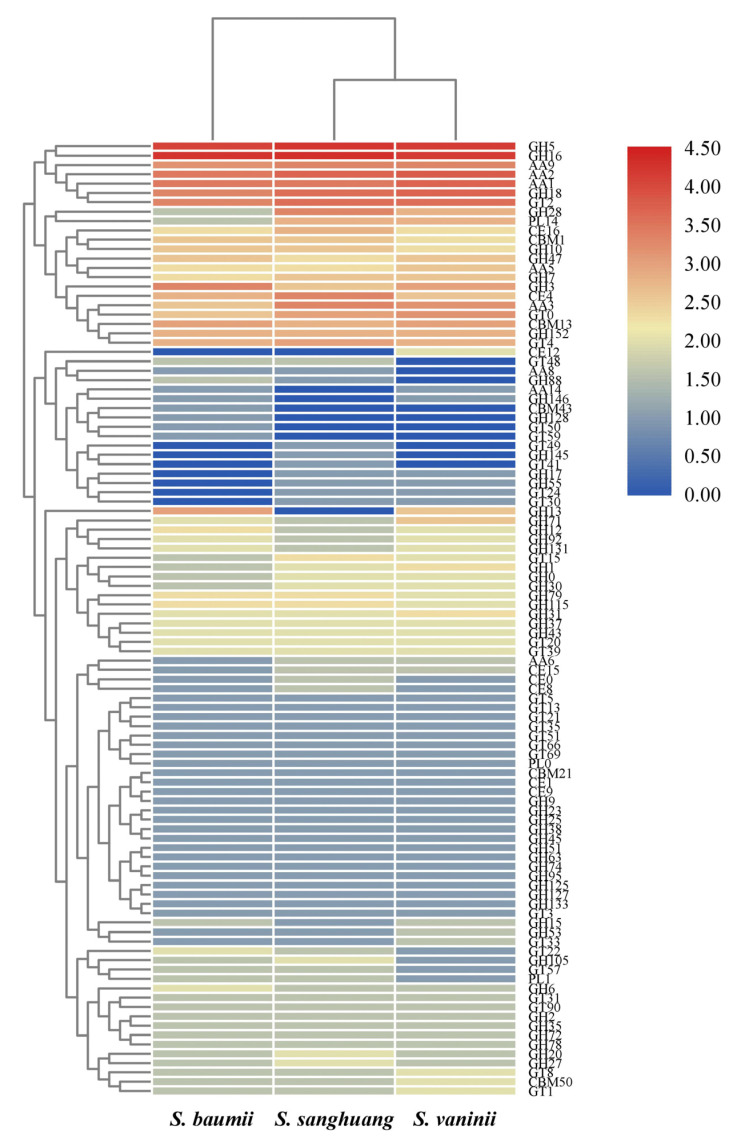
Heatmap of CAZyme families found in *Sanghuangporus baumii*, *S. sanghuang* and *S. vaninii*. The x and y axes represent species and CAZyme families, respectively. Boxes are colored by the log values of gene numbers encoding CAZyme families. The box color from dark blue to dark red indicates the increase in gene numbers encoding CAZyme families.

The total number of genes encoding CAZymes among the three species of *Sanghuangporus* were more or less similar, so was the number of genes encoding each of the six classes of CAZymes (Table 3). Only a few differences could be concluded at the level of gene families encoding CAZymes (Figure 4). According to the number of genes belonging to different families of CAZymes, *S. sanghuang* had a more similar strategy for the utilization of woody substrates with *S. vaninii* than with *S. baumii* (Figure 4).

The number of genes encoding CYPs had no significant difference at the class level from the three species of *Sanghuangporus* (Table 3).

## 4. Discussion

### 4.1. Accurate Link of Whole Genome Sequencing to Sanghuangporus sanghuang

“Sanghuang” is a kind of typical medicinal macrofungi in traditional Chinese medicines, and has been recognized to possess well medicinal properties [3]. Fifteen species in *Sanghuangporus* are considered to be “*Sanghuang*” with *S. sanghuang* as the best medicinal species [3,6,7]. After the accurate species identification, the current study presented the first whole genome sequencing of *S. sanghuang*. Notably, the genome sequence of *S. sanghuang* was previously published [17], but it was actually generated from *S. vaninii* [7]. In the case the distinct differences of these two species in medicinal properties [21], the mislabeled species name *S. sanghuang* to the genome sequenced from *S. vaninii* would, undoubtfully, restrict the accurate medicinal application of these two species. This further confirmed the opinion that systematics is crucial for the traditional Chinese medicinal studies and the industry of macrofungi [20].

### 4.2. Medicinal Properties of Sanghuangporus

During the period in which our world is endangered by a pandemic virus, Covid-19, a multidisciplinary international collaboration is crucial to win the war against Covid-19 and other viruses [37]. From this perspective, secondary metabolites from medicinal macrofungi will definitely provide a new chance to face the challenge resulted from various viruses [38]. Of the medicinal metabolites, terpenoids and polysaccharides are two major groups of chemical compounds with multiple pharmacological activities in *Sanghuangporus* [3] such as in other medicinal macrofungi [39].

Of the gene clusters of secondary metabolites, filamentous fungi have more T1PKS and NRPS gene clusters, while basidiomycetes have more TS gene cluster [40]. Correspondingly, the three species of *Sanghuangporus* had more TS gene cluster that participates in the biosynthesis of terpenoids than other gene clusters (Table 3). Triterpenoids were extracted from *S. sanghuang* with either an addition of fungal polysaccharide elicitors [41] or ultrasonic waves, and showed a high antioxidant activity [42]. The genes involved in the conserved MVA pathway of the terpenoid backbone biosynthesis participate in synthesizing triterpenoids. *Sanghuangporus baumii*, *S. sanghuang* and *S. vaninii* share a similar gene type and gene number annotated in the MVA pathway (Table 3), which indicates a similar medicinal potential of triterpenoids in these three species. More studies on the biological properties of triterpenoids in *S. baumii* and *S. vaninii* should be performed. Besides the genes involved in the biosynthesis of triterpenoids, several genes involved in the biosynthesis of sesquiterpenoids were also annotated from *S. sanghuang*. This is the first report that the potential biosynthesis pathway of other kinds of terpenoids except triterpenoids exists in *Sanghuangporus*. Noteworthily, sesquiterpenoids were extracted from an undescribed Kenyan species of *Sanghuangporus* and showed antibacterial, antifungal and cytotoxic activities [43]. Taken together, sesquiterpenoids may be a common group of biologically active secondary metabolites in *Sanghuangporus*.

Polysaccharides are the most studied metabolites in macrofungi [5]. Various molecular weights, branching configurations, conformations and chemical modifications of polysaccharides provide the structural basis for their diverse biological activities, such as antitumor, antioxidant, anti-inflammation and immunomodulation [44]. These medicinal functions were also revealed from *Sanghuangporus* [3]. We, for the first time, identified a pathway of polysaccharide biosynthesis in *S. sanghuang*, viz. the starch and sucrose metabolism pathway, in which 41 genes were involved (Figure S3, Table S2). Similarly, 40 and 46 genes involved in the pathway of polysaccharide biosynthesis were revealed from *S. baumii* and *S. vaninii*, respectively (Table 3). The higher gene numbers of *Sanghuangporus* involved in the pathway of polysaccharide biosynthesis than those in other medicinal pathways suggest that polysaccharides may be the main medicinal metabolites of *Sanghuangporus*. However, further experiments are needed to test this hypothesis.

Dissimilar to terpenoids and polysaccharides, flavonoids are rarely focused on in macrofungi, partially due to their lower content occupying the proportions of total metabolites. However, comparing with other macrofungi, *S. sanghuang* had a relatively higher content of flavonoids, showing antioxidant, antiproliferative and anti-microbial activities [45]. Seven putative genes involved in the biosynthesis of flavonoids were identified from *S. vaninii*, although with those in the model plant *Arabidopsis* as references [17]. Here, the reannotation from genome sequences via the KEGG pathway indicated that the gene number involved in the pathway of flavonoid biosynthesis from *S. sanghuang* was comparable with those from *S. baumii* and *S. vaninii* (Table 3). Therefore, flavonoids in *Sanghuangporus* deserve much more attention in medicinal studies.

A previous study showed that *S. sanghuang* possesses better medicinal properties of antitumor, antioxidant and anti-inflammation than *S. baumii* and *S. vaninii* [21], the current genomics analyses identified that *S. sanghuang* has much more T1PKS gene clusters in comparison with these two species (Table 3). These gene clusters may endow *S. sanghuang* with these advantageous medicinal properties. In addition, the differential expressions of genes related to medicinal properties may also contribute to the various medicinal properties within *Sanghuangporus*. Transcriptomic and metabolic data could help to further solve this issue.

### 4.3. Clues to Artificial Cultivation of Sanghuangporus sanghuang

One of the reasons that restrict the medicinal application of macrofungi in a large scale is the lack of available mature fruiting bodies. One practical solution to this restriction is artificial cultivation [39]. Genomics information may provide clues to facilitating the cultivation of *S. sanghuang*.

Although *S. vaninii* has been widespread cultivated, the mating type in *Sanghuangporus* has still not been clarified. From the genome sequence of the monokaryon strain MS2, one *HD* gene surrounded by *β-fg* and *mip* genes was identified. The linkage of *HD*, *β-fg* and *mip* genes in the *MAT-A* locus is highly conserved in *Basidiomycota* [46]. However, the arrangement of flanking genes to *HD* genes can be different. For example, normally, the *mip* gene locates in the upstream of *HD* genes, while the *β-fg* gene in the downstream of *HD* genes [46], whereas both *mip* and *β-fg* genes locate in the upstream of *HD* genes in *Moniliophthora roreri* [47] and in the downstream in *H. erinaceus* [14]. In *S. sanghuang*, the *β-fg* and *mip* genes, respectively, are located in the upstream and downstream of the *HD* gene. The differentiated arrangement of *HD*, *β-fg* and *mip* genes may have resulted from genome rearrangement [46]. Regarding the *MAT-B* locus, it locates on contig two and is far away from the *MAT-A* locus locating on contig eight. Given that the above identified *MAT-A* and *MAT-B* loci are not linked in the genome of the monokaryon strain MS2, it is reasonable to conclude that *S. sanghuang* has a tetrapolar heterothallic mating system. More monokaryon strains of *S. sanghuang* need to be genome sequenced in association with mating tests to further confirm this conclusion.

To investigate the utilization strategy of different woody substrates for cultivation, the CAZymes of *S. sanghuang* were analyzed in comparison with *S. baumii* and *S. vaninii*. In all these three species, genes encoding GH5, GH16, AA9, AA2, AA1 and GH18 were relatively abundant (Figure 4). GH5, known as the cellulase family A, has a large variety of degradation specificities and has been frequently identified in metagenomes from different ecological niches [48]. GH16 is involved in the degradation of xyloglucan [49]. AA9 is a family of copper-dependent lytic polysaccharide monooxygenases that are an important oxidative enzyme for the decomposition of polysaccharides such as cellulose [50]. AA2 contains class II lignin-modifying peroxidases. AA1 is a family of multicopper oxidases that degrade lignin using diphenols as electron donors and oxygen as the acceptor [51]. GH18 can break down all forms of chitin at varying rates [52]. The enrichment of genes encoding GH5, GH16, AA9, AA2, AA1 and GH18 in *Sanghuangporus* highlights their importance in lignocellulose degradation and, thus, these genes can be considered to be potential core genes for the nutritional utilization. Moreover, these genes can be used as candidates to develop molecular markers for breeding high-yield cultivars of *Sanghuangporus*. Compared with *S. baumii* and *S. vaninii*, certain families of CAZymes, such as CE16, GH28 and CE4, are encoded by more genes in *S. sanghuang* (Figure 4). This indicates that *S. sanghuang* also has its own nutritional strategy, besides that shared in *Sanghuangporus*.

Even though the knowledge of the mating type and CAZymes can promote the breeding and cultivation of *S. sanghuang*, the technological problems of how to fruit basidiocarps still need to be overcome. In future, comparative transcriptomics at the stages of primordium and fruiting body formations between *S. sanghuang* and *S. vaninii* will reveal the key genes involved in these processes and, thus, help cultivate *S. sanghuang*.

### 4.4. Specification in Sanghuangporus

Several well-known medicinal macrofungi, viz. *G. lucidum*, *G. sinense*, *S. commune*, *T. camphoratus* and *W. cocos*, were separated from *Sanghuangporus* and mostly from each other in the current phylogeny (Figure 2A). This phylogenetic relationship is reasonable, because some other traits in fungi also present multiple origins from their ancestors [53,54,55]. The phenomenon of a convergent evolution is considered to be resulted from a latent homology that means the presence of a conserved genetic toolkit for the same function across distinct clades [53]. Dissimilar to traits of yeast form, multicellularity and symbiosis, medicinal properties may not be essential to the macrofungal life cycle; therefore, it is hard to find what its genetic toolkit is across a large evolutionary scale.

At the generic level, the so-called toolkit may be present in the ancestor of *Sanghuangporus*, which diversified 15.39 Mya in Cenozoic (Figure 2A). The time point is later than that of 30.85 Mya estimated on the basis of multiloci [56]. This could have been caused by the inclusion of less species of *Sanghuangporus* in the current analysis. However, the whole genome sequence indeed provided a possibility for understanding the dynamic of the speciation event. Generally, the speciation of *S. baumii*, *S. sanghuang* and *S. vaninii* may be driven mainly by translocation and translocation plus inversion of genome sequences from their common ancestor. In the case that the three species of *Sanghuangporus* share overlapping emergence times with their specific hosts (*Syringa*, *Morus* and *Populus* 13, 23 and 17 Mya, respectively) [33], the driven dynamic of fungal speciation may be to adapt to the different lignocellulose composition and defense mechanisms of their own specific hosts.

The current phylogenomics analysis revealed a large-scale loss of gene families in fungi (Figure 2A). Indeed, this kind of loss has been identified from all life forms and considered to be a prevalent evolutionary force [57]. Because the genes and gene clusters related to the nutritional strategy and medicinal properties have no extremely significant difference within *S. baumii*, *S. sanghuang* and *S. vaninii*, we speculate that the expansion and contraction of gene families, especially those involved in a rapid evolution, contribute a lot to the host specificity of *Sanghuangporus* species. CYPs are a superfamily of monooxygenases and are involved in many biological processes across various life forms. However, compared with those in mammals and plants, the functions of CYPs in fungi are not well known [58,59]. In the current case, 25 of 121 CYPs could not be classified into any known class (Table 3). It was reported that the unique CYPs in basidiomycetous biotrophic plant pathogens could make the fungi adaptive to various ecological niches [60]. Therefore, the 25 unique CYPs involved in unknown biological processes may facilitate the adaptation of *S. sanghuang* to its host plants. In addition, the similar proportion and number of CYPs with an undetermined classification were also annotated in *S. baumii* and *S. vaninii* (Table 3) and, likewise, these CYPs may also be related to the adaptation of these two fungal species to their host plants. Future evidence from transcriptomics and functional experiments will determine whether the gene families involved in expansion and contraction and encoding unique CYPs are really related to the adaptation to hosts in the speciation of *Sanghuangporus*.

## 5. Conclusions

In general, the whole genome sequence of *S. sanghuang* represented by the monokaryotic strain MS2 was generated for the first time. The current genomic analyses indicated that *S. sanghuang* has a potential tetrapolar heterothallic mating system, and has its special nutritional strategy and advantageous medicinal properties compared with *S. baumii* and *S. vaninii*. Moreover, the species diversification of *Sanghuangporus* may be driven by the translocation and translocation plus inversion of genome sequences, while the expansion and contraction of gene families may contribute to the host specificity of *Sanghuangporus* species. Hopefully, the comprehensive understanding of the *S. sanghuang* genome will pave the way for its future roles in pharmacological and industrial applications. Future transcriptomic and metabolic data will further facilitate the accurate application of *S. sanghuang*.

## Figures and Tables

**Table 1:** Information of genome sequences used in genome analyses.

Phylum	Species	Voucher	BioProject ID	Number of Contigs	Max Length of Contig (Mb)	N50 Length of Contig (Mb)
*Ascomycota*	*Neurospora crassa*	OR74A	PRJNA13841	21	9.80	6.00
	*Tuber melanosporum*	Mel28	PRJEA38847	398	2.79	0.64
*Basidiomycota*	*Agaricus bisporus*	H97	PRJNA61005	13	3.34	2.55
	*Auricularia subglabra*	TFB-10046 SS5	PRJNA60553	1531	2.21	0.49
	*Fomitiporia mediterranea*	MF3/22	PRJNA56107	1412	9.60	4.29
	*Ganoderma lucidum*	G.260125-1	PRJNA71455	82	4.83	1.39
	*Ganoderma sinense*	ZZ0214-1	PRJNA42807	69	4.26	2.26
	*Hericium alpestre*	DSM 108284	PRJNA521006	3534	0.21	0.02
	*Heterobasidion irregulare*	TC 32-1	PRJNA46703	15	3.59	2.57
	*Phellinidium pouzarii*	DSM 108285	PRJNA521454	1776	0.26	0.04
	*Pleurotus ostreatus*	PC9	PRJNA647232	17	4.83	3.50
	*Polyporus arcularius*	HHB13444	PRJNA196048	2540	0.31	0.05
	*Porodaedalea pini*	BCRC 35384	PRJNA380037	220	2.99	0.57
	*Pyrrhoderma noxium*	FFPRI411160	PRJNA377805	13	4.53	2.74
	*Rickenella mellea*	SZMC22713	PRJNA334780	848	2.92	0.36
	*Sanghuangporus baumii*	821	PRJNA304358	217	1.38	0.27
	*Sanghuangporus sanghuang*	MS2	PRJNA731629	26	2.98	2.06
	*Sanghuangporus vaninii*	Kangneng	PRJNA564179	37	3.67	2.02
	*Schizophyllum commune*	H4-8	PRJNA32757	36	7.10	2.55
	*Schizopora paradoxa*	KUC8140	PRJNA239088	1291	1.82	0.12
	*Taiwanofungus camphoratus*	S27	PRJNA244959	360	2.22	1.03
	*Trametes versicolor*	FP-101664 SS1	PRJNA56097	283	5.13	2.88
	*Wolfiporia cocos*	MD-104	PRJNA52943	348	5.11	2.54

**Table 2:** De novo genome assembly and features of *Sanghuangporus sanghuang*.

Contig	Characteristic	Genome	Characteristic
Total number	26	Genome assembly (Mb)	33.34
Total length (Mb)	33.34	Number of protein-coding genes	8278
N50 length (Mb)	2.06	Average length of protein-coding genes (bp)	1597
Max length (Mb)	2.98	Repeat size (Mb)	1.25
Min length (kb)	80.44	Transposable elements (Mb)	0.96
Coverage (%)	99.98	tRNA (bp)	7836
GC content (%)	48.04		

**Table 3:** Comparative genomics analyses of *Sanghuangporus baumii*, *S. vaninii* and *S. sanghuang*.

Characteristic		*S. baumii*	*S. sanghuang*	*S. vaninii*
Genome structure	Genome size (Mb)	31.64	33.34	34.52
	Number of contigs	339	26	56
	N50 length of contig (Mb)	0.18	2.06	2.02
	Protein-coding genes	8455	8278	11,310
	GC content (%)	47.25	48.04	47.95
Gene involved in the pathway of medicinal metabolites	Terpenoid backbone biosynthesis	15	14	15
	Polysaccharide biosynthesis	40	41	46
	Flavonoid biosynthesis	6	8	7
Gene encoding CAZymes	CBM	39	38	38
	CE	15	23	18
	GH	155	162	160
	GT	54	62	61
	PL	5	9	8
	AA	45	52	53
	Sum	313	346	338
Gene encoding cytochromes P450	B-class P450	1	0	0
	Cytochrome P450	8	12	9
	E-class P450, CYP2D	1	1	1
	E-class P450, group I	68	66	80
	E-class P450, group IV	7	8	14
	P450, CYP52	5	3	6
	Pisatin demethylase-like	4	6	2
	Undetermined	18	25	24
	Sum	112	121	136
Gene cluster of secondary metabolites	TS	12	10	11
	T1PKS	1	4	1
	NRPS	3	1	3
	Other molecules	2	1	1
	Sum	18	16	16

## Data Availability

Publicly available datasets were analyzed in this study. All newly generated sequences were deposited in GenBank (https://www.ncbi.nlm.nih.gov/genbank/).

## Supplementary Materials

The following are available online at https://www.mdpi.com/article/10.3390/jof7100787/s1. File S1: information of 8278 predicted protein-coding genes in *Sanghuangporus sanghuang*, Figure S1: pathway of terpenoid backbone biosynthesis in *Sanghuangporus sanghuang*, Figure S2: pathway of sesquiterpenoid and triterpenoid biosynthesis in *Sanghuangporus sanghuang*, Figure S3: pathway of polysaccharide biosynthesis (starch and sucrose metabolism) in *Sanghuangporus sanghuang*, Figure S4: genomic blocks of *Sanghuangporus baumii*, *S. sanghuang* and *S. vaninii*, Table S1: putative genes involved in the pathway of terpenoid backbone biosynthesis in *Sanghuangporus sanghuang*, Table S2: putative genes involved in the biosynthesis of polysaccharides (starch and sucrose metabolism) in *Sanghuangporus sanghuang*, Table S3: putative genes involved in the biosynthesis of uridine diphosphate glucose in *Sanghuangporus sanghuang*, Table S4: putative genes involved in the biosynthesis of flavonoid compounds in *Sanghuangporus sanghuang*.
