# Supplementary material for: The First Whole Genome Sequencing of Sanghuangporus sanghuang Provides Insights into Its Medicinal Application and Evolution
Source: J Fungi (Basel). 2021 Sep 22;7(10):787. doi: 10.3390/jof7100787 (PMC8537844; doi:10.3390/jof7100787)
Supplement: Supplementary file 1 [file jof-07-00787-s001.zip › Supplementary Materials/Supplementary Materials.pdf]

File S1. Information of 8278 predicted protein-coding genes in *Sanghuangporus sanghuang.*

Figure S1. Pathway of terpenoid backbone biosynthesis in *Sanghuangporus sanghuang*. The blue and green boxes indicate the presence of single- and double-copy of homologous genes encoding the enzymes, respectively.

Figure S2. Pathway of sesquiterpenoid and triterpenoid biosynthesis in *Sanghuangporus sanghuang*. The blue boxes indicate the presence of single-copy of homologous genes encoding the enzymes**.**

Figure S3. Pathway of polysaccharide biosynthesis (starch and sucrose metabolism) in *Sanghuangporus sanghuang*. The blue, green, yellow and orange boxes indicate the presence of single-, double-, triple- and four- to nine-copy of homologous genes, respectively.

Figure S4. Genomic blocks of *Sanghuangporus baumii*, *S. sanghuang* and *S. vaninii*. Shared blocks of collinearity are linked by pink lines, blocks of translocation by blue lines, blocks of inversion by orange lines, and blocks of translocation and inversion by green lines.

Table S1. Putative genes involved in the pathway of terpenoid backbone biosynthesis in *Sanghuangporus sanghuang*.

**Gene name and definition**	**Enzyme code**	**KO term**	**Gene ID**
FDPS; farnesyl diphosphate synthase	2.5.1.1, 2.5.1.10	K00787	A1964
mvaD; diphosphomevalonate decarboxylase	4.1.1.33	K01597	A6841
FNTA; protein farnesyltransferase/geranylgeranyltransferase type-1 subunit alpha	2.5.1.58, 2.5.1.59	K05955	A6934
STE24; STE24 endopeptidase	3.4.24.84	K06013	A1904
HMGCR; hydroxymethylglutaryl-CoA reductase (NADPH)	1.1.1.34	K00021	A1041
atoB; acetyl-CoA C-acetyltransferase	2.3.1.9	K00626	A2749
GGPS1; geranylgeranyl diphosphate synthase, type III	2.5.1.1, 2.5.1.10, 2.5.1.29	K00804	A3347
mvaK2; phosphomevalonate kinase	2.7.4.2	K00938	A3410
E2.3.3.10; hydroxymethylglutaryl-CoA synthase	2.3.3.10	K01641	A2231
idi; isopentenyl-diphosphate Delta-isomerase	5.3.3.2	K01823	A5810
hexPS; hexaprenyl-diphosphate synthase	2.5.1.82, 2.5.1.83	K05355	A7458
FCLY; prenylcysteine oxidase/farnesylcysteine lyase	1.8.3.5, 1.8.3.6	K05906	A6316
FNTB; protein farnesyltransferase subunit beta	2.5.1.58	K05954	A1834
SRT1; ditrans, polycis-polyprenyl diphosphate synthase	2.5.1.87	K11778	A4415

Table S2. Putative genes involved in the biosynthesis of polysaccharides (starch and sucrose metabolism) in *Sanghuangporus sanghuang*.

Gene name and definition	Enzyme code	KO term	Identity (%)	*E*-value	Gene ID
SGA1; glucoamylase	3.2.1.3	K01178	84.1	4.10E-290	A2599
HK; hexokinase	2.7.1.1	K00844	74	7.30E-211	A2696
GBE1, glgB; 1,4-alpha-glucan branching enzyme	2.4.1.18	K00700	86.8	0	A2720
E3.2.1.4; endoglucanase	3.2.1.4	K01179	64.3	1.20E-170	A2968
PYG, glgP; glycogen phosphorylase	2.4.1.1	K00688	90.2	0	A3794
TSL1, TPS3; trehalose 6-phosphate synthase complex regulatory subunit	--	K22337	77.6	0	A3936
AMY, amyA, malS; alpha-amylase	3.2.1.1	K01176	73.5	7.70E-216	A4004
CBH1; cellulose 1,4-beta-cellobiosidase	3.2.1.91	K01225	75.1	1.40E-208	A4217
CBH1; cellulose 1,4-beta-cellobiosidase	3.2.1.91	K01225	74.7	3.00E-209	A4220
GYS; glycogen synthase	2.4.1.11	K00693	92.5	0	A4243
beta-glucosidase	3.2.1.21	K01188	80.4	0	A4328
CBH2, cbhA; cellulose 1,4-beta-cellobiosidase	3.2.1.91	K19668	80.1	3.90E-164	A4452
AMY, amyA, malS; alpha-amylase	3.2.1.1	K01176	76.6	1.10E-241	A4496
1,3-beta-glucan synthase	2.4.1.34	K00706	89.9	0	A4637
bglX; beta-glucosidase	3.2.1.21	K05349	86.1	0	A4980
AMY, amyA, malS; alpha-amylase	3.2.1.1	K01176	54.6	9.60E-117	A5429
TREH, treA, treF; alpha,alpha-trehalase	3.2.1.28	K01194	88.7	0	A5537
otsA; trehalose 6-phosphate synthase	2.4.1.15, 2.4.1.347	K00697	88.4	8.90E-282	A5558

Endoglucanase	3.2.1.4	K01179	81.5	7.70E-91	A5669
Endoglucanase	3.2.1.4	K01179	88.5	1.30E-185	A5687
bglX; beta-glucosidase	3.2.1.21	K05349	76.9	0	A5895
malZ; alpha-glucosidase	3.2.1.20	K01187	75.1	0	A6004
UGP2, galU, galF; UTP--glucose-1-phosphate uridylyltransferase	2.7.7.9	K00963	88.7	1.70E-257	A6356
TPS; trehalose 6-phosphate synthase/phosphatase	2.4.1.15, 3.1.3.12	K16055	83.6	0	A7139
beta-glucosidase	3.2.1.21	K01188	79	1.70E-250	A7142
CBH2, cbhA; cellulose 1,4-beta-cellobiosidase	3.2.1.91	K19668	88.5	1.50E-192	A7429
glucan 1,3-beta-glucosidase	3.2.1.58	K01210	73.4	2.00E-173	A7446
glucan 1,3-beta-glucosidase	3.2.1.58	K01210	64.8	3.00E-165	A7447
glucan 1,3-beta-glucosidase	3.2.1.58	K01210	68.7	1.60E-171	A7449
GYG1, GYG2; glycogenin	2.4.1.186	K00750	64	0	A7574
AGL; glycogen debranching enzyme	2.4.1.25, 3.2.1.33	K01196	82.3	0	A8029
TREH, treA, treF; alpha,alpha-trehalase	3.2.1.28	K01194	78.1	8.30E-308	A0242
HK; hexokinase	2.7.1.1	K00844	90.5	4.70E-263	A0434
1,3-beta-glucan synthase	2.4.1.34	K00706	90.2	0	A0471
beta-glucosidase	3.2.1.21	K01188	89.1	4.10E-258	A0544
IMA, malL; oligo-1,6-glucosidase	3.2.1.10	K01182	62.3	1.20E-223	A1140
pgm; phosphoglucomutase	5.4.2.2	K01835	88.9	4.20E-294	A2275

CBH1; cellulose 1,4-beta-cellobiosidase	3.2.1.91	K01225	83.3	2.40E-229	A2394
CBH1; cellulose 1,4-beta-cellobiosidase	3.2.1.91	K01225	83.3	2.40E-229	A2395
CBH1; cellulose 1,4-beta-cellobiosidase	3.2.1.91	K01225	79.1	2.80E-222	A2404
GPI, pgi; glucose-6-phosphate isomerase	5.3.1.9	K01810	90.9	3.40E-299	A3598

Table S3. Putative genes involved in the biosynthesis of uridine diphosphate glucose in *Sanghuangporus sanghuang*.

Gene name and definition	Enzyme code	KO term	Identity (%)	*E*-value	Gene ID
1,3-beta-glucan synthase	2.4.1.34	K00706	90.2	0	A0471
1,3-beta-glucan synthase	2.4.1.34	K00706	89.9	0	A4637
ADP-ribosylation factor GTPase-activating protein 1	--	K12492	76.6	3.90E-197	A2453
endo-1,3(4)-beta-glucanase	3.2.1.6	K01180	45.6	1.20E-81	A7815
endo-1,3(4)-beta-glucanase	3.2.1.6	K01180	48.2	1.50E-86	A7766
endo-1,3(4)-beta-glucanase	3.2.1.6	K01180	62.8	2.80E-185	A7704
endo-1,3(4)-beta-glucanase	3.2.1.6	K01180	47.7	2.60E-78	A6709
endo-1,3(4)-beta-glucanase	3.2.1.6	K01180	50.8	1.00E-102	A6508
endo-1,3(4)-beta-glucanase	3.2.1.6	K01180	48.6	8.70E-87	A6470
endo-1,3(4)-beta-glucanase	3.2.1.6	K01180	48.2	3.30E-101	A4205
endo-1,3(4)-beta-glucanase	3.2.1.6	K01180	41.4	4.80E-66	A4142
endo-1,3(4)-beta-glucanase	3.2.1.6	K01180	59.9	3.60E-103	A3963
endo-1,3(4)-beta-glucanase	3.2.1.6	K01180	58.5	8.30E-192	A2927
endo-1,3(4)-beta-glucanase	3.2.1.6	K01180	52.5	2.70E-168	A2872
endo-1,3(4)-beta-glucanase	3.2.1.6	K01180	66.1	5.20E-89	A2858
endo-1,3(4)-beta-glucanase	3.2.1.6	K01180	49.2	6.00E-86	A2219
endo-1,3(4)-beta-glucanase	3.2.1.6	K01180	49.4	6.80E-157	A1496
endo-1,3(4)-beta-glucanase	3.2.1.6	K01180	41.1	2.80E-71	A1374
endo-1,3(4)-beta-glucanase	3.2.1.6	K01180	47.9	1.50E-50	A1344
endo-1,3(4)-beta-glucanase	3.2.1.6	K01180	64.9	3.30E-195	A1087
endo-1,3(4)-beta-glucanase	3.2.1.6	K01180	43.6	1.40E-81	A1034
endo-1,3(4)-beta-glucanase	3.2.1.6	K01180	54.2	5.00E-195	A0107
GTPase-activating protein BEM2	--	K19844	70.1	0	A5627
GTPase-activating protein SAC7	--	K19845	60	1.60E-171	A5513
GTPase-activating protein SST2	--	K19838	87	0	A2703
Hexokinase	2.7.1.1	K00844	70.7	7.60E-188	A3346
Hexokinase	2.7.1.1	K00844	90.5	4.70E-263	A2696
Hexokinase	2.7.1.1	K00844	74	7.30E-211	A0434
phosphoglucomutase	5.4.2.2	K01835	49.5	5.80E-133	A5826
phosphoglucomutase	5.4.2.2	K01835	88.9	4.20E-294	A2275

Ran GTPase-activating protein 1	--	K14319	85	1.00E-177	A7607
Rho GTPase-activating protein 1	--	K18470	71	1.50E-239	A2059
Rho-type GTPase-activating protein ½	--	K19839	76.8	5.90E-275	A6706
UTP--glucose-1-phosphate uridylyltransferase	2.7.7.9	K00963	88.7	1.70E-257	A6356

Table S4. Putative genes involved in the biosynthesis of flavonoid compounds in *Sanghuangporus sanghuang*.

Gene name and definition	Enzyme code	KO term	Identity (%)	*E*-value	Gene ID
isoflavonoid 7-O-beta-apiosyl-beta-glucosidase	3.2.1.161	K20852	75.6	4.10E-231	A0544
isoflavonoid 7-O-beta-apiosyl-beta-glucosidase	3.2.1.161	K20852	43.7	2.10E-112	A7142
isoflavonoid 7-O-beta-apiosyl-beta-glucosidase	3.2.1.161	K20852	41.7	6.00E-111	A7157
chalcone 461beta-O-glucosyltransferase	2.4.1.286	K21372	46.6	2.60E-32	A4702
chalcone 486beta-O-glucosyltransferase	2.4.1.286	K21372	56.5	4.30E-238	A6287
Chalcone-flavanone isomerase	5.5.1.6	K01859	84	2.30E-276	A6232
Chalcone-flavanone isomerase	5.5.1.6	K01859	84	2.30E-276	A6233
Chalcone-flavanone isomerase	5.5.1.6	K01859	84	2.30E-276	A7868
flavonol L-rhamnosyltransferase	2.4.1.159	K22772	46.6	2.60E-32	A4702
flavonol L-rhamnosyltransferase	2.4.1.159	K22772	56.5	4.30E-238	A6287
