# Supplementary figures and images for: The First Whole Genome Sequencing of Sanghuangporus sanghuang Provides Insights into Its Medicinal Application and Evolution
Source: J Fungi (Basel). 2021 Sep 22;7(10):787. doi: 10.3390/jof7100787 (PMC8537844; doi:10.3390/jof7100787)

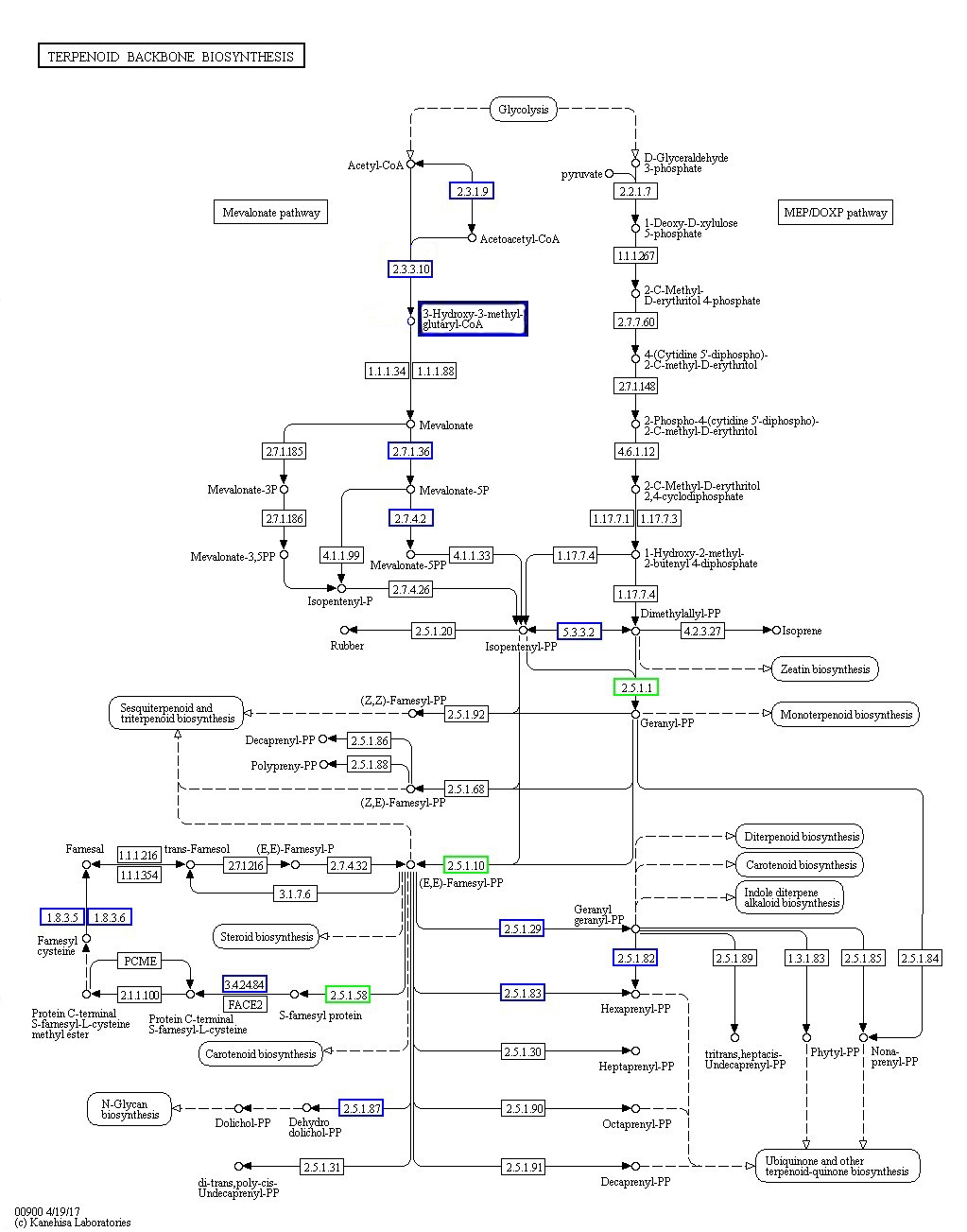

Supplement: Supplementary file 1 [file jof-07-00787-s001.zip › Supplementary Materials/Figure S1.jpg]

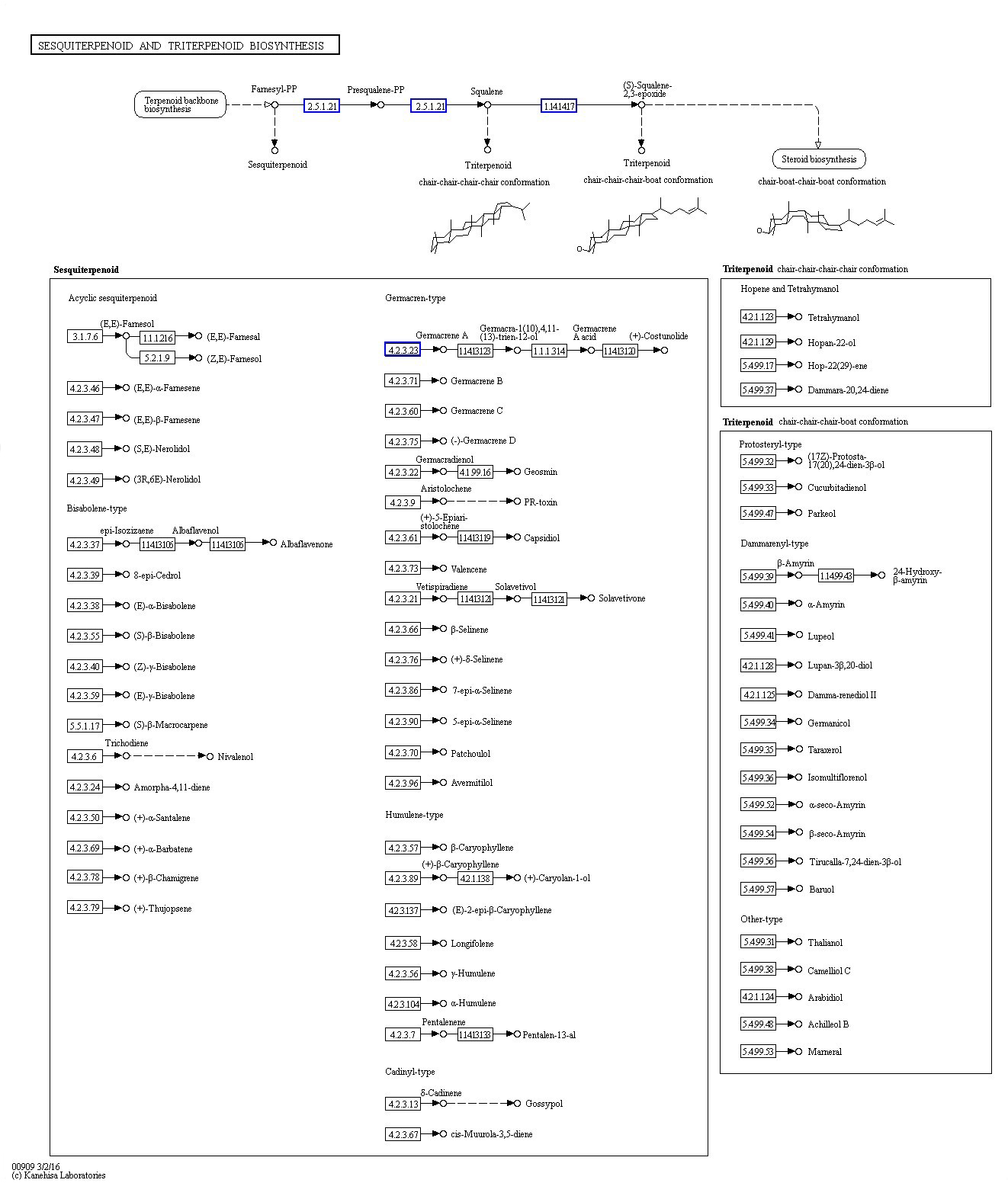

Supplement: Supplementary file 1 [file jof-07-00787-s001.zip › Supplementary Materials/Figure S2.jpg]

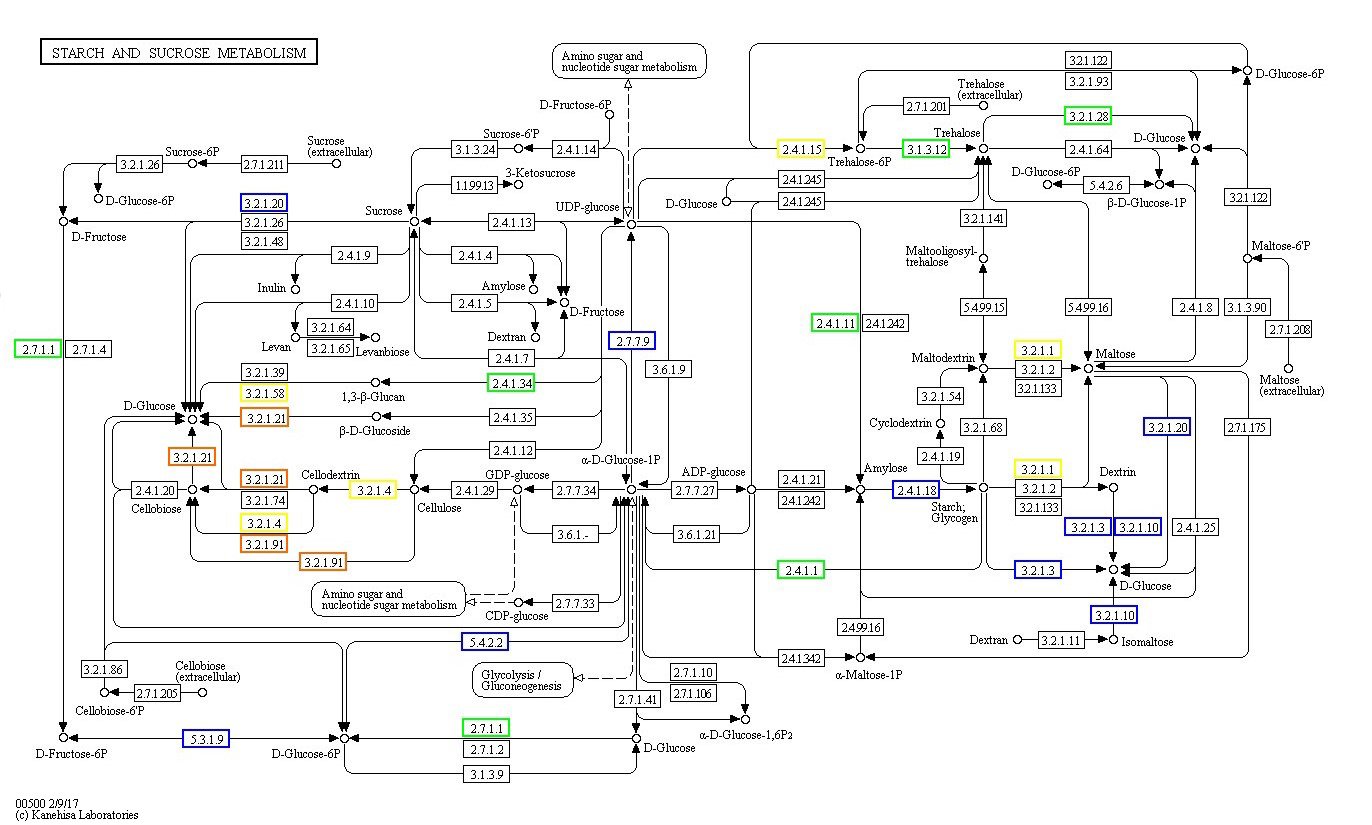

Supplement: Supplementary file 1 [file jof-07-00787-s001.zip › Supplementary Materials/Figure S3.jpg]

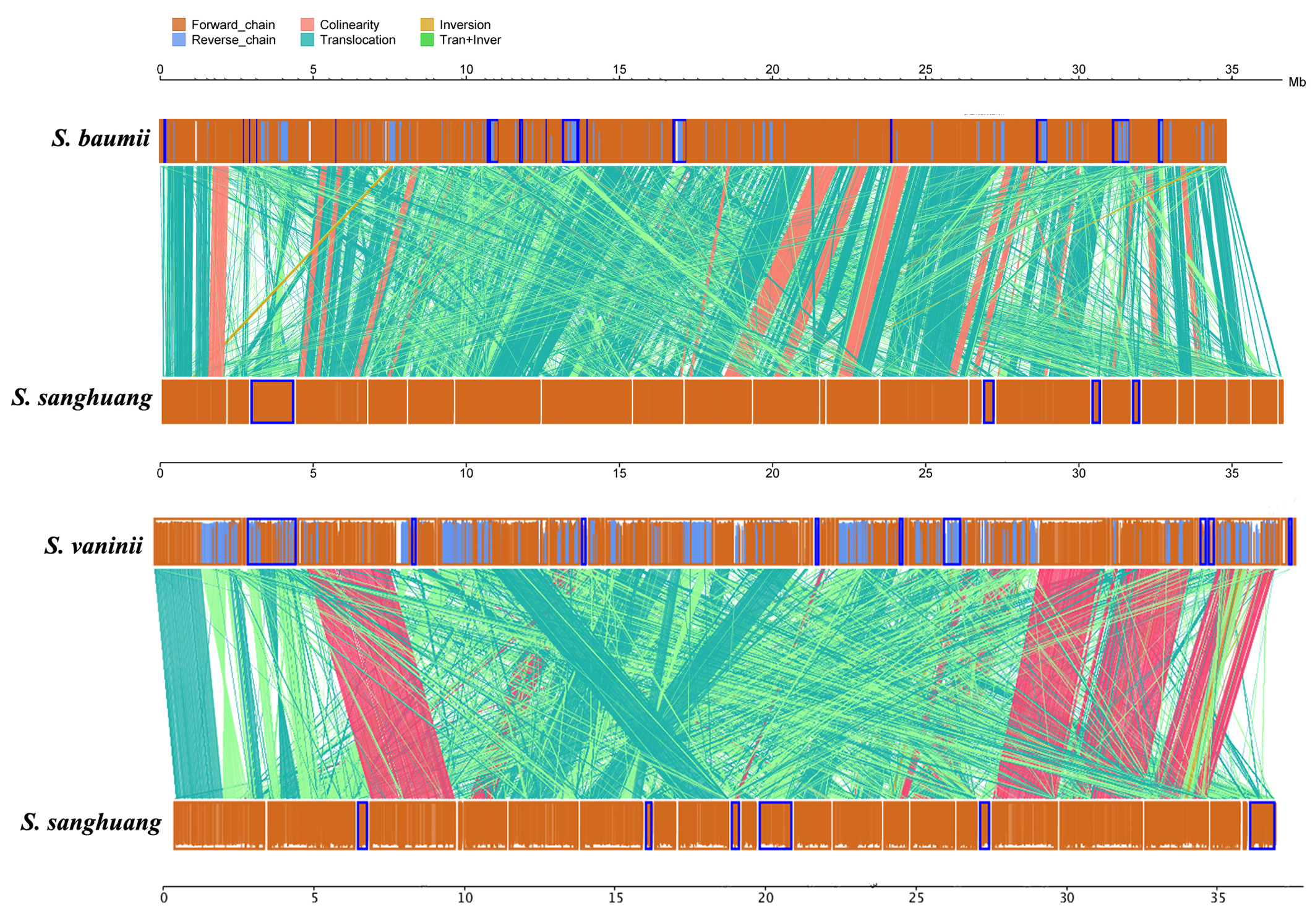

Supplement: Supplementary file 1 [file jof-07-00787-s001.zip › Supplementary Materials/Figure S4.jpg]
